# Testing Attention Restoration in a Virtual Reality Driving Simulator

**DOI:** 10.3389/fpsyg.2019.00250

**Published:** 2019-02-11

**Authors:** Marica Cassarino, Marta Maisto, Ylenia Esposito, Davide Guerrero, Jason Seeho Chan, Annalisa Setti

**Affiliations:** ^1^School of Applied Psychology, University College Cork, Cork, Ireland; ^2^School of Allied Health, Health Research Institute, Faculty of Education and Health Sciences, University of Limerick, Limerick, Ireland; ^3^Department of Psychology, Seconda Università degli Studi di Napoli, Naples, Italy

**Keywords:** attention restoration, driving simulator, virtual environment, driving behavior, mental fatigue, cognitive load

## Abstract

**Objectives:** Attention Restoration Theory (ART) suggests that walking or being in natural settings, as opposed to urban environments, benefits cognitive skills because it is less demanding on attentional resources. However, it is unclear whether the same occurs when the person is performing a complex task such as driving, although it is proven that driving through different road environments is associated with different levels of fatigue and may engage attention differently. The present study investigated whether exposure to rural vs. urban road environments while driving would affect attentional capacity in young people after the drive, in line with the classic ART paradigms.

**Methods:** We asked 38 young participants to complete the Sustained Attention to Response Task (SART) before and after being exposed to a rural or urban road in a virtual reality environment while driving in a full vehicle immersive driving simulator. Changes in SART performance based on environmental exposure where explored in terms of target sensitivity, accuracy, reaction times, and inverse efficiency. We analyzed potential road type effects on driving speed and accuracy. Possible effects of driving on attention were tested by comparing the sample performance to that of a control group of 15 participants who did not drive and sat on the passenger seat instead.

**Results:** Exposure to rural or urban road environments in the driving sample was not associated with any significant changes in attentional performance. The two exposure groups did not differ significantly in terms of driving behavior. Comparisons between the driving sample and the control group controlling for age indicated that participants who drove were more accurate but slower at the SART than those who were passengers.

**Conclusion:** The present study does not support the hypothesis that a short drive in a natural setting may promote attention restoration as compared to an urban setting. Methodological considerations as well as recommendations for future research are discussed.

## Introduction

Increasingly, research has demonstrated that road characteristics can impact both driving behavior and the activities the driver will undertake once arrived to destination (Antonson et al., 2009; Keay et al., 2009; Calvi, 2015; Murphy and Greene, 2016; Cassarino and Murphy, 2018). Urban roads present higher road complexity than rural roads and can impose more cognitive demands on the driver (Murphy and Greene, 2016). Higher cognitive demands translate not only into less safe driving, but also into poorer cognitive performance after the drive (Murphy and Greene, 2016). These findings support the idea that environmental situations that are perceptually complex (e.g., presenting visual clutter) engage more attentional resources and are thus more cognitively fatiguing (Lavie et al., 2004). Complementing this hypothesis, encouraging evidence suggests that the impact of road characteristics on drivers’ attentional resources may depend on the presence of natural elements. A review on roadside features and driving safety (Wilde, 2010) suggested that green scenery on the road can have restorative effects on attention. Similarly, a recent review indicated that roadside vegetation can reduce drivers’ stress and frustration (Van Treese et al., 2017). This research is informed by Attention Restoration Theory (ART, Kaplan and Kaplan, 1989; Kaplan, 1995); ART suggests that natural environments are more restorative for attention than urban settings because engaging bottom-up involuntary attention (defined as “soft fascination”) while “freeing-up” top-down directed attentional resources (Kaplan and Berman, 2010; Bratman et al., 2012; Berto, 2014). Several behavioral studies support ART by showing that even a brief exposure to natural vs. urban settings, either through walking or seeing images, can relieve from the attentional fatigue caused by a cognitive task completed prior to the environmental exposure (Hartig et al., 2003; Berto, 2005; Berman et al., 2008). Supportive evidence has come from neuroimaging as well (Martínez-Soto et al., 2013; Bratman et al., 2015; Chen et al., 2016), although recent systematic reviews have shown that restorative effects are small (de Keijzer et al., 2016; Ohly et al., 2016).

While most studies on attention restoration have used walking as a form of real-life exposure to nature, very little is known about cognitive restoration in relation to driving, which requires monitoring of the road, and therefore a certain level of attentional engagement. If nature engages bottom-up attention only, one should expect that driving in roads with natural elements, such as rural roads, should be associated with less attentional fatigue than driving on urban roads. In line with this hypothesis, an experimental study used a pre- and post- design where participants were mentally stressed before being exposed to video-tapes of either highway vegetation or roads with man-made material of 5-min duration (Cackowski and Nasar, 2003); an assessment of mental stress after exposure found higher tolerance to frustration in participants who viewed natural rather than urban roads. However, no studies to our knowledge have tested ART while driving using the classic experimental paradigm described above (i.e., changes in attention).

In the present study, we used a simulated driving paradigm to test the hypothesis that driving in an urban or rural virtual environment would differentially impact on attentional fatigue after completing a demanding cognitive task (Sustained Attention to Response Task or SART, Robertson et al., 1997), as shown in previous studies on nature and sustained attention (Berto, 2005). The SART is a measure of attentional capacity as well as the ability to inhibit unwanted responses for a prolonged time; it has been used in previous investigations of ART as a way to mentally fatigue participants *before* exposure to natural or urban scenes and to measure attention restoration *after* exposure (Berto, 2005). In the present study, a pre-post design was employed, whereby participants performed the SART task before and after either a rural or urban drive. Assuming that rural roads are more restorative (i.e., less cognitively demanding) than urban roads due to the presence of green, we hypothesized that driving through a rural rather than urban environment after having been mentally fatigued would be associated with improvements in attentional performance at the end the drive. Given the very high usage of cars in our society, investigating the impact of road nature on drivers’ cognitive abilities has important implications for enhancing our understanding of how road characteristics influence cognitive performance.

## Materials and Methods

### Participants

In line with Berto (2005), a total of 38 participants (Mean age = 22.1, *SD* = 3.43; 44% female) were recruited through convenience sampling among undergraduate and graduate students at University College Cork, Ireland. Participants were randomly assigned to an urban or rural environmental exposure (*n* = 19 in each group). Half of the participants (*n* = 19) were fully licensed drivers with an average of 5.5 years of driving experience (*SD* = 3.24), whereas the other half (*n* = 19) included individuals with no full license and mean driving experience of 2.3 years (*SD* = 3.81). All participants read and signed a consent form prior to data collection in accordance with the Declaration of Helsinki. Ethical approval for the study was received by the School of Applied Psychology Ethics Committee, University College Cork. All participants read an information sheet briefing on the aims of the study and all were asked to read and sign a written consent form prior to participation in the study. No vulnerable populations were included in the study.

### Design

A 2 × 2 mixed between-within design was employed, with the participants’ performance at SART, (assessed pre- vs. post- exposure to virtual reality environments in a full vehicle driving simulator) as the within-subjects factor; and Environment type (urban vs. rural) as the between-subjects factor.

### Material and Apparatus

#### Sustained Attention to Response Task (SART)

**FIGURE 1:**
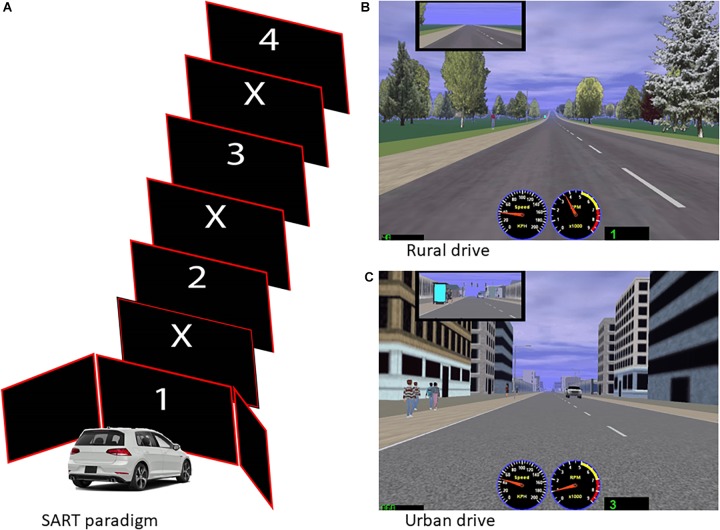
**(A)** Sustained Attention to Response Task (SART) paradigm: A sequence of digits was viewed by the participants while sitting in the vehicle; digits appeared in the central project screen of the simulator. Participants completed the SART before and after driving in the simulated environment, consisting of a computerized graphic depicting a rural **(B)** or urban **(C)** scenario.

The SART is an experimental paradigm used to measure sustained attention (Robertson et al., 1997). In this task, participants viewed a random sequence of digits (1–9) appearing on the central projector screen of the simulator, while sitting in the vehicle (see Figure 1A). A computer keyboard was placed on the participant’s lap and they were asked to press the spacebar as quickly as possibly at the appearance of each digit, except for the digit three. The numbers appeared on the center screen of the simulator. The task was programmed in E-Prime 2.0 software.

Before the experimental block there were 18 practice trials so that participants were accustomed to the task and apparatus. The experimental block consisted of 252 trials (28 of each digit between one and nine) presented in one of five semi-randomly assigned fonts in the range of 12–29 centimeters. In the test trial, the target stimulus (i.e., the number 3) appeared 28 times, while the remaining 224 digits were non-lures. Digits appeared on the screen every 1,125 ms, for the duration of 200 ms, followed by a 900 ms mask, which was a diagonal cross contained within a 29-centimeter ring. Both the digits and the mask were white against a black background. Instructions on how to complete the task were showed on the computer screen prior to the appearance of both the practice and the test trial.

#### Virtual Reality Environments

The driving simulator we utilized is considered to be a medium-level driving simulator (not high as it is not placed on a steward platform). It consists of a full-size Volkswagen Polo vehicle with manual transmission which has all vehicle controls available including functional speedometer and tachometer. The vehicle faces 3 large projection screens and has LCD screens in the wing mirrors and a rear projection screen which can be viewed through the rear-view mirror. The vehicle is equipped with 7.1 Dolby surround sound, enabling the creation of a more immersive environment with engine sounds as well as noises from other road users such as beeping or harsh braking. The simulator is housed in a dark, cool room with black-out blinds, black walls and a fan to provide airflow. In the adjacent control room, the experimenter can monitor the participants’ progress. The simulator uses STISIM 400W software (STISIMdrive.com) which allows for flexible programming various driving environments.

A computerized graphic rural drive and an urban drive were designed for the study, as shown in Figure 1B,C. The rural drive (Figure 1B) presents a road surrounded by trees (isolated or in groupings) and fields; while the urban drive presents the same kind of road surrounded by some pedestrians and buildings either low rise commercial or tall commercial or residential buildings (Figure 1C). All drives were designed to minimize the occurrence of features which are known to cause simulator sickness, such as curves or sudden stops (Classen et al., 2011). Similar to previous studies on ART, the scenarios were pilot tested for perceived pleasantness and restorative potential with a separate group of participants utilizing the Attention Restoration Scale (Hartig et al., 1997). Participants were assessed for motion sickness through a questionnaire before and after the drive, closely monitored for any signs of sickness, offered regular breaks and reminded that they could withdraw from the study at any point.

Driving behavior was recorded in terms of average speed, standard deviation from the average speed, lane position, and mistakes (including, number of occurrences of red-light tickets, speed excess, collisions, and road lane excursions).

### Procedure

Participants were first introduced to the vehicle and its controls. They were then given a 10-min practice drive to become accustomed to the responsiveness of the vehicle. This practice drive consisted of a mix between urban and rural environments. Once participants were comfortable the practice drive was stopped, and they were presented with the first SART task. Once they completed the SART task they were randomly assigned to drive in the rural or urban environment. During the drive, which lasted 10 min, participants were asked to maintain a speed of approximately 60 Km/h. The duration of the test drive was based on Berto (2005), who found that 10 min of viewing images of natural scenes was enough for participants to experience restoration. Also, previous studies have demonstrated differences in cognitive performance between drivers exposed to different scenarios after a 10–12 min test drive (Murphy and Greene, 2016). This duration was also chosen to avoid potential discomfort for the participants. The participants were then asked to complete the SART again (Session 2), after which they filled a short demographic questionnaire.

### Statistical Analyses

Participants’ performance at the SART was analyzed in terms of d-prime (d’: a measure of signal detection sensitivity, calculated as the standardized difference (z-scores) between the proportion of correct responses on non-lures minus the proportion of incorrect responses on lures), overall mean accuracy (proportion of correct responses on lures and non-lures), mean accuracy on non-lures (pressing the bar), accuracy on lures (not pressing the bar when number three appears), reaction times (in milliseconds) of correct responses (related to pressing the bar in the presence of a non-lure), and inverse efficiency, a measure of speed-accuracy trade-off calculated as the ratio of reaction times over accuracy on non-lures (Bruyer and Brysbaert, 2011). Comparisons between the two exposure groups in terms of gender were conducted using Chi-square test and potential differences in age and driving experience were investigated via an independent samples *t*-test. These comparisons were carried out to decide whether demographic status or driving experience should be included in the subsequent analyses as covariates. A 2 × 2 mixed-design ANOVA was conducted with Environment (rural vs. urban) as the between-subjects factor, and SART (pre- vs. post-drive) as the within-subjects factor to investigate effects of environmental exposure on changes in attentional performance pre- and post-drive. *Post hoc* comparisons were conducted via *t*-test statistics. Comparisons between exposure groups in terms of driving behavior were assessed via independent *t*-test. In addition, potential effects of driving on attention were tested through a 2 (SART session) × 2 (environmental exposure) × 2 (driving vs. passenger condition) ANOVA with Driving (driver or passenger) and Environment (urban vs. rural) as the between-subject factors, and SART (pre- vs. post-drive) as the within-subjects factor. We conducted a test of normality on the ANOVA unstandardized residuals as well as the Levene’s test of homogeneity (see Supplementary File 1); for measures that did not appear to meet the assumptions of normality, we conducted the analyses using non-parametric tests and found no differences in results (see Supplementary File 1).

## Results

### Environmental Exposure Effects on Attention

The two exposure groups (*n* = 19 in each group) did not differ significantly in terms of gender ($\chi_{1}^{2}$ = 0.11, *p* = 0.74), age (*t_36_* = -0.42, *p* = 0.67) or driving experience (*t_36_* = 0.16, *p* = 0.87).

The 2 × 2 mixed-design ANOVA indicated no significant interaction between environmental exposure and SART pre- and post-drive for any of the measures of interest, as shown in Table 1.

**Table 1:** Interaction between environmental exposure and SART session - driving sample.

Measure	*F*(1,36)	*P*-value
d’	0.96	0.76
Total accuracy	0.05	0.82
Accuracy on lures	0.003	0.96
Accuracy on non-lures	0.06	0.81
Reaction times	0.004	0.95
Inverse efficiency	0.008	0.93


*F refer to a 2 × 2 mixed design ANOVA.*

There was a main effect of environmental exposure for the measure of d’ (*F_1,36_* = 4.18, *p* = 0.048, μ^2^ = 0.11), with participants in the rural exposure group (*M* = 1.26, *SD* = 1.07) showing overall higher sensitivity (i.e., better performance) than the urban exposure group (*M* = 0.62, *SD* = 0.84). There was also a main effect of environmental exposure for the measure of accuracy on lures (*F_1,36_* = 4.61, *p* = 0.04, μ^2^ = 0.11), with participants in the rural group (*M* = 0.64, *SD* = 0.25) being overall more accurate than those in the urban group (*M* = 0.48, *SD* = 0.21). In both cases, however, the size of the effect was small.

We found that the driving behavior of two exposure groups did not differ significantly for any of the measures of interest: average speed (*t_35_* = 0.21, *p* = 0.84), standard deviation from average speed (*t_35_* = 0.61, *p* = 0.55), average lane position (*t_36_* = 0.03, *p* = 0.97), standard deviation from average lane position (*t_36_* = -1.71, *p* = 0.09), speed excess (*t_36_* = 0.45, *p* = 0.65), or lane excursions (*t_36_* = 1.67, *p* = 0.11).

### Testing for the Effect of Driving

As an additional check on our study, we conducted a control study whereby we recreated the same situation, but participants were not required to drive. This was included so that the act of driving could be dissociated from viewing motion. We initially recruited 24 participants (12 in the rural condition and 11 in the urban group); however, eight participants (four in each condition) did not complete the driving scenario due to motion sickness or unwillingness, leaving a final sample of 15 participants (Mean age = 31.26, *SD* = 6.69; 53.3%% female); these completed the SART before and after a 10-min exposure to the virtual environment (*n* = 8 rural vs. *n* = 7 urban road) while seating in the driver’s seat but not driving.

We ran a 2 (SART session) × 2 (environmental exposure) ANOVA for this group with exposure (urban vs. rural) as the between-subject factors, and SART (pre- vs. post-drive) as the within-subjects factor. As shown in Table 2, no interactions emerged for any of the measures of interest.

**Table 2:** Interaction between environmental exposure and SART session – control sample.

Measure	*F*(1,13)	*P*-value
d’	1.62	0.23
Total accuracy	1.32	0.27
Accuracy on lures	0.04	0.85
Accuracy on non-lures	0.91	0.36
Reaction times	1.36	0.26
Inverse efficiency	0.48	0.49


*F refer to a 2 × 2 mixed design ANOVA.*

Similarly, no main effects of environmental exposure emerged. A main effect of session was noted for total accuracy (*F_1,13_* = 5.22, *p* = 0.04, μ^2^ = 0.27) with an overall small improvement from baseline (*M* = 0.63, *SD* = 0.16) to post-exposure (*M* = 0.68, *SD* = 0.16).

We then pooled together the data (*N* = 53) from the two samples (driving, *n* = 38; non-driving, *n* = 15), and ran a 2 (SART session) × 2 (environmental exposure) × 2 (driving vs. passenger condition) ANOVA with Driving (driver or passenger) and Environment (urban vs. rural) as the between-subject factors, and SART (pre- vs. post-drive) as the within-subjects factor. As the driving group was older than the non-driving group (*t_51_* = 6.59, *p* = 0.000, Cohen’s *d* = 1.72), we included age as a covariate in the ANOVA.

Controlling for age, we found no significant interactions (not shown); a main effect of driving condition emerged for all measures except inverse efficiency (d’: *F_1,48_* = 49.93, *p* = 0.000, μ^2^ = 0.48; total accuracy: *F_1,48_* = 65.41, *p* = 0.000, μ^2^ = 0.52; accuracy on lures: *F_1,48_* = 6.85, *p* = 0.01, μ^2^ = 0.12; accuracy on non-lures: *F_1,48_* = 50.02, *p* = 0.000, μ^2^ = 0.45; reaction times: *F_1,48_* = 31.18, *p* = 0.000, μ^2^ = 0.38). Specifically, participants who drove were significantly more accurate and slower at the SART than those in the control group (i.e., not driving) both before and after exposure, and independent of exposure condition.

Only in the case of accuracy on non-lures, a main effect of exposure also emerged (*F_1,48_* = 5.34, *p* = 0.03, μ^2^ = 0.05), with participants exposed to the rural environment being overall more accurate (*M* = 0.92, *SD* = 0.11) than those exposed to the urban environment (*M* = 0.88, *SD* = 0.21); however, the effect size of environmental exposure was smaller than that of driving.

## Discussion

The present study tested attention restoration theory (ART) by investigating the potential effects on attention of exposure to urban or rural roads while driving. Overall our findings do not support the hypothesis that driving in a rural natural environment is more restorative of attentional fatigue. Our study is novel, as to our knowledge no other studies have employed the specific experimental paradigm of our study, particularly utilizing a driving simulator. The utilization of the driving simulator paradigm may be the reason why our results are in contrast with existing evidence of changes in sustained attention after exposure to images of urban vs. natural scenes (Berto, 2005) or after a walk in an urban or green environment (Hartig et al., 2003; Berman et al., 2008).

We investigated whether potential differences in driving behavior could have influenced these results, however the two exposure groups drove with similar speed (as requested, with few infractions) and accuracy. We also tested whether the driving task could influence the effect of being exposed to urban or rural environments by re-running the experiment in a sample of participants who seated in the car but did not drive, as a further control condition. No restorative effects were noted in this group either, while a small practice effect was found. When comparing the two driving groups (driving vs. passenger), we found that participants who drove were more accurate but slower at the SART than those who were passengers, showing more conservative performance in both sessions. These differences did not appear to depend on sample characteristics such as age. Notably, the passenger group showed an overall improvement in accuracy independent of exposure, which might indicate a practice effect and possibly that the virtual immersion served as a resting interval for both exposure groups (i.e., not driving might have led the participants to not engage enough with the virtual environment to generate restorative effects). However, this interpretation of the results needs to be considered with caution, as the differences between driving conditions at baseline did not depend on the type of environmental exposure and might be related either to a selection bias which we were unable to capture or to the potential effects of the different types of instructions provided to participants at the beginning of the experiment (i.e., one group was asked to drive while the other sat in the car, and this might have created different expectations as well as different levels of engagement with the experiment).

One could argue that completing the SART was an easy task for our sample, and therefore did not cause attentional fatigue. However, the performance of our sample was worse than that of Berto (2005), who used the same cognitive task in a sample of similar age. While this comparison supports the idea that our participants were mentally fatigued by the SART, future studies might assess other measures of mental fatigue other than the SART.

A limitation of our study is the realism of the urban and rural drives, which are clearly a simulation and therefore less rich than real natural scenes in terms of soft fascination features. Nonetheless, these scenarios were pilot tested for perceived pleasantness and restorative potential with a separate group of participants. When considering the study by Berto (2005), which also utilized the SART, it is important to note two critical differences: Firstly, the restorative scenes used in the driving simulator were not photographs of real environments. It is possible that a removal from a realistic scene does not provide scenery-related restoration. Secondly, in all situations the scenery was moving (to give the sense that the car was in motion). This is unlike previous studies where participants viewed static images for 10 min. Linked to this, it is possible that the short duration of the test drive (10 min) might have been insufficient to generate restorative effects; however, restorative effects of nature have been demonstrated after short exposures (Berto, 2005), and previous studies using simulated drives have shown effects on cognitive performance for durations similar to that of our study (Murphy and Greene, 2016).

While the small sample size of each subgroup, as well as the imbalanced number of participants in the two driving conditions, limited the power of our analyses, it is worth noting that the effect sizes were very small; in addition, previous studies on attention restoration have shown effects with samples comparable to the present study.

In light of our results, the present study shows that driving or being a passenger in a simulated drive with no particularly challenging situations does not overall determine a different load on attention after the drive. Of course, a different scenario could be envisaged whereby the drives are very demanding, for e.g., an urban drive with pedestrians suddenly crossing the road, however, such a scenario would differ substantially from the traditional ART paradigms (e.g., observation of scenes). Therefore, our study contributes to the current knowledge about cognitive restoration and natural settings by indicating that attention restoration may not occur when the individual is on a moving vehicle, therefore, potentially less engaged in soft fascination.

## Data Availability Statement

The raw data supporting the conclusions of this manuscript will be made available by the authors, without undue reservation, to any qualified researcher.

## Author Contributions

MC, JC, and AS were major contributors in writing the manuscript. MC, MM, YE, DG, JC, and AS designed the study. MM, YE, and DG conducted the data collection and participated in the data analysis. MC, JC, and AS were major contributors in the data analysis. All authors read and approved the final manuscript.

## Conflict of Interest Statement

The authors declare that the research was conducted in the absence of any commercial or financial relationships that could be construed as a potential conflict of interest.
