# Supplementary material for: Testing Attention Restoration in a Virtual Reality Driving Simulator
Source: Front Psychol. 2019 Feb 11;10:250. doi: 10.3389/fpsyg.2019.00250 (PMC6378321; doi:10.3389/fpsyg.2019.00250)
Supplement: Supplementary file 1 [file Table_1.DOCX]

**Driving Cognitive Restoration paper**

**Supplementary File 1**

**Driving sample**

| Supplementary Table 1 | |
| --- | --- |
| *Normality test of ANOVA unstandardized residuals* | |
| Measure | Shapiro-Wilk  *p*-value |
| D-prime S1 | .09 |
| D-prime S2 | .55 |
| Accuracy Total S1 | .001 |
| Accuracy Total S2 | .000 |
| Accuracy Lures S1 | .17 |
| Accuracy Lures S2 | .23 |
| Accuracy Non-lures S1 | .000 |
| Accuracy Non-lures S2 | .000 |
| Reaction time S1 | .64 |
| Reaction time S2 | .05 |
| Inverse efficiency S1 | .63 |
| Inverse efficiency S2 | .02 |

| Supplementary Table 2 | |
| --- | --- |
| *ANOVA Homogeneity test* | |
| Measure | Levene’s test  *p*-value |
| D-prime S1 | .32 |
| D-prime S2 | .67 |
| Accuracy Total S1 | .65 |
| Accuracy Total S2 | .76 |
| Accuracy Lures S1 | .23 |
| Accuracy Lures S2 | .69 |
| Accuracy Non-lures S1 | .49 |
| Accuracy Non-lures S2 | .92 |
| Reaction time S1 | .05 |
| Reaction time S2 | .75 |
| Inverse efficiency S1 | .05 |
| Inverse efficiency S2 | .73 |

Nonparametric analysis (Wilcoxon Signed ranked test by condition) for measures not meeting assumption of normality:

- Accuracy Total: no significant changes in rural exposure (*Z* = -0.85, *p* = .39) or urban exposure (*Z* = -0.51, *p* = .62)
- Accuracy Non-lures: no significant changes in rural exposure (*Z* = -0.87, *p* = .38) or urban exposure (*Z* = -0.06, *p* = .95)
- Inverse efficiency: no significant changes in rural exposure (*Z* = -1.08, *p* = .27) or urban exposure (*Z* = -1.82, *p* = .07)

**Passenger sample**

| Supplementary Table 3 | |
| --- | --- |
| *Normality test of ANOVA unstandardized residuals* | |
| Measure | Shapiro-Wilk  *p*-value |
| D-prime S1 | .85 |
| D-prime S2 | .04 |
| Accuracy Total S1 | .03 |
| Accuracy Total S2 | .000 |
| Accuracy Lures S1 | .55 |
| Accuracy Lures S2 | .05 |
| Accuracy Non-lures S1 | .08 |
| Accuracy Non-lures S2 | .001 |
| Reaction time S1 | .17 |
| Reaction time S2 | .94 |
| Inverse efficiency S1 | .000 |
| Inverse efficiency S2 | .000 |

| Supplementary Table 4 | |
| --- | --- |
| *ANOVA Homogeneity test* | |
| Measure | Levene’s test  *p*-value |
| D-prime S1 | .74 |
| D-prime S2 | .57 |
| Accuracy Total S1 | .22 |
| Accuracy Total S2 | .14 |
| Accuracy Lures S1 | .36 |
| Accuracy Lures S2 | .45 |
| Accuracy Non-lures S1 | .19 |
| Accuracy Non-lures S2 | .13 |
| Reaction time S1 | .53 |
| Reaction time S2 | .57 |
| Inverse efficiency S1 | .06 |
| Inverse efficiency S2 | .08 |

Nonparametric analysis *(*Wilcoxon Sined ranked test by condition) for measures not meeting assumption of normality:

- Accuracy Total: no significant changes in rural exposure (*Z* = -1.83, *p* = .07) or urban exposure (*Z* = -1.68, *p* = .09)
- Accuracy Non-lures: no significant changes in rural exposure (*Z* = -1.33, *p* = .18) or urban exposure (*Z* = -1.69, *p* = .09)
- Inverse efficiency: no significant changes in rural exposure (*Z* = -0.86, *p* = .38) or urban exposure (*Z* = -1.33, *p* = .18)

**Pooled data**

| Supplementary Table 5 | |
| --- | --- |
| *Normality test of ANOVA unstandardized residuals* | |
| Measure | Shapiro-Wilk  *p*-value |
| D-prime S1 | .12 |
| D-prime S2 | .95 |
| Accuracy Total S1 | .000 |
| Accuracy Total S2 | .000 |
| Accuracy Lures S1 | .83 |
| Accuracy Lures S2 | .28 |
| Accuracy Non-lures S1 | .000 |
| Accuracy Non-lures S2 | .000 |
| Reaction time S1 | .08 |
| Reaction time S2 | .03 |
| Inverse efficiency S1 | .000 |
| Inverse efficiency S2 | .000 |

| Supplementary Table 6 | |
| --- | --- |
| *ANOVA Homogeneity test (Exposure, driving, controlling for age)* | |
| Measure | Levene’s test  *p*-value |
| D-prime S1 | .05 |
| D-prime S2 | .11 |
| Accuracy Total S1 | .000 |
| Accuracy Total S2 | .000 |
| Accuracy Lures S1 | .23 |
| Accuracy Lures S2 | .66 |
| Accuracy Non-lures S1 | .000 |
| Accuracy Non-lures S2 | .000 |
| Reaction time S1 | .000 |
| Reaction time S2 | .000 |
| Inverse efficiency S1 | .000 |
| Inverse efficiency S2 | .003 |

Nonparametric analysis *(*Wilcoxon Signed ranked test by condition) for measures not meeting assumption of normality

| Supplementary Table 7 | |  |  |  |
| --- | --- | --- | --- | --- |
| *Nonparametric analysis S2 – S1* | |  |  |  |
|  | Rural exposure | | Urban exposure | |
|  | Driver | Passenger | Driver | Passenger |
| Measure | Z (*p*-value) | Z (*p*-value) | Z (*p*-value) | Z (*p*-value) |
| Accuracy Total | -0.85 (.39) | -1.83 (.07) | -0.51 (.61) | -1.68 (.09) |
| Accuracy Non-lures | -0.88 (.38) | -1.33 (.18) | -0.06 (.95) | -1.69 (.09) |
| Reaction times | -1.28 (.19) | -0.94 (.35) | -1.77 (.07) | -1.06 (.28) |
| Inverse Efficiency | -1.08 (.27) | -0.86 (.38) | -1.81 (.07) | -1.33 (.18) |
